# Deciding as Intentional Action: Control over Decisions

**DOI:** 10.1080/00048402.2014.971035

**Published:** 2014-10-17

**Authors:** Joshua Shepherd

**Affiliations:** ^a^ University of Oxford

**Keywords:** control, deciding, mental action, practical deliberation, skill

## Abstract

Common-sense folk psychology and mainstream philosophy of action agree about decisions: these are under an agent's direct control, and are thus intentional actions for which agents can be held responsible. I begin this paper by presenting a problem for this view. In short, since the content of the motivational attitudes that drive deliberation and decision remains open-ended until the moment of decision, it is unclear how agents can be thought to exercise control over what they decide at the moment of deciding. I note that this problem might motivate a non-actional view of deciding—a view that decisions are not actions, but are instead passive events of intention acquisition. For without an understanding of how an agent might exercise control over what is decided at the moment of deciding, we lack a good reason for maintaining commitment to an actional view of deciding. However, I then offer the required account of how agents exercise control over decisions at the moment of deciding. Crucial to this account is an understanding of the relation of practical deliberation to deciding, an understanding of skilled deliberative activity, and the role of attention in the mental action of deciding.

## 1. Introduction

Towards the end of World War II, Harry Truman—then President of the United States—faced a choice: drop atomic bombs on Japan, or send American troops to invade. One option involved the certain death of thousands of Japanese women and children. Another involved the certain death of thousands of American troops. Truman decided to drop the bombs.

At the end of game four of the 1987 NBA Finals, Magic Johnson—then point guard for the Los Angeles Lakers—found himself with an open shot. Johnson was a bit far from the basket, however, and there were six seconds still on the clock: too much time. Johnson decided not to shoot (four seconds later, and much closer to the basket, he would take his shot—a hook shot). Not as momentous a decision as Truman's, but not easy either.

As folk psychologists, we regard practical decisions—decisions about what to do—as expressions of agency. In momentous and mundane contexts, we consider the decisions agents make to be intentional actions, and in part because of this we hold agents responsible for what they decide to do. Is this common-sense view justified?

According to much recent philosophy of action, yes. Many uphold common-sense folk psychology by maintaining that practical decisions are momentary intentional actions of intention formation [Frankfurt 1988: 174–6; Kane 1996: 24; McCann 1998: 163; Searle 2001: 94; Clarke 2003: 3–27; Mele 2003: 197–202]. Some philosophers go further than this. Thomas Pink, for example, identifies the will with ‘a capacity for decision-making or intention formation’ [1996: 16]. Gary Watson holds that deciding is ‘an ubiquitous instance of agency’, and that ‘a human being who never engaged in such activity would be an agent only in a very truncated sense’ [2004: 126]. R. Jay Wallace maintains the following [1999: 637]:

intentions, decisions, and choices are things we do, primitive examples of the phenomenon of agency itself. It is one thing to find that one wants some chocolate cake very much, or that its odor reminds one of one's childhood in Detroit, quite another to resolve to eat a piece. The difference, I would suggest, marks a line of fundamental importance, the line between the passive and the active in our psychological lives.

This is congenial to much work on free will and moral responsibility, which tends to take decisions to be of central import [Campbell 1957; van Inwagen 1989; Kane 1996; Pink 1996; Mele 2005]. As Derk Pereboom has put it, ‘The view that responsibility for decisions is especially important is driven by the sense that responsibility is fundamentally a matter of control, a kind of control agents would have primarily over their decisions’ [2001: xxi]. If decisions were not intentional actions, this view and the attention given to decisions in this literature would seem quite misguided.

In section 2, I present a problem for an actional view of deciding. I argue that, since the content of the motivational attitudes that drive deliberation and decision remains open-ended until the moment of decision, it is unclear how agents can be thought to exercise direct control over their decisions. And if agents cannot exercise direct control over their decisions—that is, if agents cannot exercise control over what is decided at the moment of deciding—then a nonactional view of deciding begins to look attractive.

In sections 3–5, I develop a solution to the problem: an account of how agents exercise control over what they decide at the moment of deciding. Crucial to this account is an understanding of skilled deliberative activity, and the role of attention in the skilled mental action of deciding.

## 2. A Control Problem for Deciding

As we have seen, many take decisions to be momentary intentional actions of intention formation. Alfred Mele explicates the view as follows [2010: 44–5]:

Deciding to A is not to be confused with any process that issues in deciding to A, including, for example, deliberation about what to do … And deciding to A, as I conceive of it, does not precede the onset of the intention to A formed in the act of deciding. Instead, what it is to decide to A is to form—actively—an intention to A. The intention arises in that momentary intention-forming action, not after it.

In virtue of what are such decisions intentional? On Mele's widely adopted view, practical decisions are intentional in part because of the causal work of a mental state extrinsic to the decision itself—an intention to decide what to do [Mele 2003: ch. 9].

Thus understood, decisions are an odd sort of intentional action (as Mele himself notes). Typically, the intentions that are relevant to intentional actions initiate, sustain, and guide action. When Magic drove to the basket, he formed an intention to shoot a hook shot. As he acted, he had a definite idea of what to do, of how his action was to unfold, of what success was supposed to look like. The intentionality of Magic's action—his making the hook shot—is explained in part by the guiding role of his intention.

It is not transparent how the intentions relevant to practical decisions might play the same sort of guiding role. For the content of these intentions is open-ended in a unique way. Before deciding, agents rarely if ever intend to decide to A. In most cases, the formation of such an intention would seem superfluous; why not simply intend to A?[Fn fn0001] ^1^Mele [2003: 202–5] offers a more detailed argument for this thought. Rather than intend to decide to A, in most cases agents intend to decide what to do (i.e. whether to A or to B or to C). Before he decided not to shoot, Magic had no definite idea of what to do, of how his action (that is, the decision he had to make) was to unfold, of what success was supposed to look like. Perhaps troublingly, before he decided to bomb Japan, neither did Truman. In a recent paper on deciding, Jenann Ismael offers an apt metaphor [2012: 160]:

think of an attempt to follow the path in sand created by your own footsteps … you cannot follow a path created by your own footsteps. You have to chart your own course. There is no danger of straying from the path, but there is also nothing there to guide your footsteps.

This is a control problem. For other types of intentional actions, it is natural to think of the control an agent possesses as that set of abilities that enables an agent to bring behaviour to match the content of relevant intentions [Shepherd 2014]. The content of intentions constrains the exercise of the abilities, and gives us a way to mark out those abilities that constitute control over a given action. Regarding decisions, however, the content of the relevant intention is not specific enough.[Fn fn0002] ^2^This is not to say that the content of the intention is limited to the very general plan to decide what to do. In section 3, I discuss some of the ways in which the context of the decision involves multiple practical constraints, and it is plausible that some of these constraints will explicitly restrict the range of options the agent takes to be available. Thus, an intention to decide what to do will at times be better described as an intention to decide amongst some restricted range of available options. It is thus difficult to see how an intention to decide what to do could give an agent guidance regarding the specific decision she makes.

Brief discussion of a recently proffered theory of rational deciding will bring the problem into sharper relief. As Franz Dietrich and Christian List [2013] show, the practical decision an agent is disposed to make in a given circumstance can be represented as a function from available alternatives to choices. The choice function is induced by a preference order over available alternatives. And the preference order—which is represented by a simple weighing relation that holds between combinations of alternatives—is dependent on the set of motivating reasons that are relevant to the available alternatives. As Dietrich and List explicate the relation, a motivating reason is a proposition that, if true of an alternative Q, may influence the agent's actual preference for Q in relation to other alternatives [2013: 107].

So far, so good. As Dietrich and List note, however, an agent's own deliberative processes have the potential to change the set of motivating reasons that influence her preference order over available alternatives [2013: 122–3]. Perhaps, for example, visualizing one alternative brings to light previously unrecognized properties of that alternative, or perhaps attention to one option activates previously dormant thoughts of yearning or disgust. The resulting picture is as follows [2013: 126]:

at any time, the agent is in a particular psychological state, represented by his or her set of motivating reasons in relation to the given alternatives, which, jointly with the agent's weighing relation, determines his or her preference order. This preference order then induces a choice function, which encodes how the agent would choose from any concrete set of alternatives. By implication, a change in the agent's set of motivating reasons can bring about not only a change in his or her preference order, but also a change in the choice function and thus in the resulting choice disposition.

As I discuss in more detail below, it is natural to conceive of deliberation as an attempt to solve some practical problem. One way to do so would be to examine one's preferences for alternatives and pick the most preferred alternative. But if deliberation itself can change an agent's preferences, then we want to know more about how an agent implements the intention to decide what to do in deliberation and at the moment of deciding. How does such implementation qualify as an exercise of control? When should the agent stop deliberating? At what point should she trust that she has enough information, and that her assessment of the information she has is adequate?

Two ways out of this control problem suggest themselves. First, one might hope to solve the problem by changing the account of practical deciding. Towards that end, consider Hugh McCann's view: according to McCann, the intentionality of deciding requires no prior motivational attitude such as an intention to decide what to do. Rather, the intentionality of deciding is intrinsic—‘a nonrelational and essential feature of the act of deciding itself’ [1998: 163]. As McCann has it, ‘When I decide, I intend to decide, and I intend to decide exactly as I do. That is, I intend to progress to having precisely the objective that is put in place by my decision’ [ibid.].

Might we build an account of control over decisions by appealing to McCann's intention to decide exactly as one does? It is difficult to see how. McCann aims to explain the intentionality of deciding by appeal to an intention to decide exactly as one does. But since no prior attitude need play an explanatory role here, it becomes mysterious whether or how an agent can exercise control over the formation of that intention. Regarding control over decisions, McCann's proposal is unpromising.

Second, one might hope to understand control over decisions by reference to some supplementary motivational attitude. According to this thought, an agent exercises control over a decision D by exercising control in service of a motivational attitude M relevant to D, where M is distinct from an intention to decide what to do or an intention to decide exactly as one does. What motivational attitude might fit this role? The most plausible options are variations of an attitude David Velleman [1992] made famous—the desire to act according to reasons.

After all, it is a compelling thought that an agent whose practical decisions accord with the reasons she recognizes and endorses is an agent in control.[Fn fn0003] ^3^Dietrich and List seem to think that the malleability of the relevant set of motivating reasons offers an agent some measure of control: ‘An agent can deliberately interrogate him- or herself about which propositions are genuine normative reasons for him or her in relation to some alternatives, and thereby exercise some influence over which reasons come to motivate him or her’ [2013: 120]. However, in the absence of an account of what guides such a process, it is unclear why the changing of preference orders by way of deliberate interrogation should be thought to be under an agent's control. So let us briefly consider the prospects for variations of this attitude, which include desires, intentions, or policies to decide to do what is best, or what is right, or whatever maximizes or promotes P, where P is some endorsed value or good.

These attitudes are unpromising as candidates for solving our problem. The reason is simple: their content remains open-ended in the problematic way. Exercising control in service of a desire to decide to do what is best provides no guidance regarding what is best. To find this out, the agent must deliberate—she must figure out what, in the context, she judges it best to do. But, as we saw in the previous section, it is plausible that deliberation itself is capable of altering an agent's preferences. It is true, of course, that this is not exactly the original decision problem. According to some philosophers of action, agents sometimes form judgments about what it is best to do and then, *akratically*, decide to do something else [Mele 2012]. So there is arguably a kind of control—sometimes called self-control, or *enkrateia*—that agents possess and exercise over one part of some of their decisions. This is control exercised in service of a judgment about what it is best to do, and against forms of competing motivation to decide otherwise.

We want to know whether agents exercise control in deciding. Noting that agents sometimes exercise self-control in forming intentions consistent with judgments about what it is best to do is of little help in this connection, for at least two reasons. First, given the importance to the decisions that follow of judgments about what it is best to do, we want to know whether agents can possess and exercise control over the judgment. So a similar problem recurs, this time regarding the judgment.

Second, whatever the relation between judgments about what it is best to decide and decisions, it is unlikely that these judgments will deliver a satisfying account of control over decisions. Assume, first, that *akratic* decisions—intentional mental actions of intention formation that run contrary to judgments about what it is best to decide—are possible. Necessarily, intentional actions are under an agent's control: an agent never acts intentionally without exercising control in so doing. Thus, on this conception, an *akratic* decision is still under the agent's control. Our problem remains.

Some hold that intentional actions contrary to one's judgment about what it is best to do are impossible [Hare 1952]. On the assumption that this is so, it becomes unclear why we should find promising the suggestion that control over decisions depends on judgments about what it is best to decide. For if such judgments are tightly connected to decisions—as they must be if it is impossible to intentionally decide contrary to them—they are ill-positioned to deliver an understanding of control over decisions. First, it is controversial whether judgments are the kinds of things over which agents can exercise direct control. But more importantly, judgments are not decisions. If one denies that intentional actions contrary to one's judgment about what it is best to do are possible, there is pressure to adopt a non-actional view of deciding.

Here a sceptical thought comes into view. Perhaps decisions are not intentional actions, but are instead passive events of intention acquisition. Galen Strawson suggests a view like this. According to Strawson [2003: 244], at least for most decisions,

[t]he movement of the natural causality of reason … to its conclusion in choice or decision is lived (by some) as action when it is really just reflex; distinctively rational reflex, to be sure, but not in any case a matter of action.[Fn fn0004] ^4^Strawson does, however, allow that for some decisions, there might be ‘some sort of genuine action of positive commitment to the decision, either at the time it is reached, or at the moment of the “passage à l'acte”’ [2003: 244].

And although he distances himself from Strawson on certain points, Wayne Wu is open to a similar view on deciding. According to Wu, though agents control their deliberative activity, it is plausible to assume that intention formations are not directly controlled by agents: ‘Judging and deciding are just the automatic culmination of an extended action’ [2013: 257].[Fn fn0005] ^5^Brian O’Shaughnessy [1980: 299–300] also maintains that decisions are not intentional actions, and it seems that his reasons for doing so stem from recognition of something like the control problem elucidated here. The control problem elucidated in this section bolsters the case for a non-actional view of deciding. Having the problem laid out before us, it begins to seem mysterious how an agent could exercise control over what she decides at the moment of deciding. This should worry proponents of an actional view of deciding. For without an understanding of how such control could be exercised, the endorsement of an actional view of deciding might depend on little more than contentious claims about phenomenology or, worse, on prior commitments regarding free will. In what follows, I offer an account of how agents exercise control over what they decide at the moment of deciding.

## 3. Practical Deliberation

In what follows, I will argue that the action of deciding is related in an important way to the activities that constitute practical deliberation (henceforth: deliberation). As I noted above, given the control problem I have elucidated about deciding, we want to know more about how an agent implements the intention to decide what to do in deliberation (and at the moment of deciding). In particular, we want to know how such implementation might qualify as an exercise of control. Getting clear on this issue as it relates to deliberation is the goal of this section and the next.

Elisabeth Camp [2009] identifies two functional properties that any system must possess if it is to engage in deliberation: systematicity and stimulus-independence.[Fn fn0006] ^6^Camp's paper is primarily about the nature of conceptual thought, which is not the same thing as practical deliberation. Issues about conceptual thought are relevant to our understanding of the nature of (especially human) practical deliberation, of course, but I utilize Camp's discussion in ways that suit my own purposes. Here I do not explicitly address the role of conceptual thought in practical deliberation. Roughly, a system possesses systematicity only if it possesses (a) the ability to represent certain particulars (e.g. events, states of affairs, properties, objects), and (b) the ability to combine and recombine these particulars to form novel thoughts. The ideal in this connection is a system that meets Gareth Evans's Generality Constraint [1982: 104]:

If a subject can be credited with the thought that a is F then he must have the conceptual resources for entertaining the thought that a is G, of every property of being G of which he has a conception.

The second crucial property is stimulus-independence. Roughly, a system possesses stimulus-independence to the degree that the exercise of its representational abilities is not under stimulus control.[Fn fn0007] ^7^Depending on the theorist, a system S's degree of stimulus-independence has been connected to S's status as a thinker [Dummett 1994], as possessing conceptual abilities [McDowell 1994], and as an agent [Hurley 2003]. Imagine an animal with the capacity, in principle, to meet the Generality Constraint. But imagine that this animal is bound by perception, in the sense that at a time t the animal is only able to represent elements of the world it perceives at t. Like a jet engine attached to a tricycle, this hypothetical animal's marvellous recombinatory abilities would rarely be put to use given its low degree of stimulus-independence. In order to put its recombinatory abilities to work, an animal needs the further ability to token and maintain representations without direct prompting from the environment.[Fn fn0008] ^8^These properties do not arise for free: plausibly, the abilities that undergird them are evolutionarily expensive. Thus, many animals exhibit these properties only in certain contexts. Susan Hurley [2003] argues, for example, that many animals exhibit these kinds of properties only in certain practical contexts, and thus that these animals occupy ‘islands of practical rationality’.


A system's possession of systematicity and stimulus-independence is bound by the representational abilities it possesses—i.e. abilities to endogenously token certain representations and to endogenously combine these representations. Let us say, then, that, for any class of representational abilities C, a system S's possession of systematicity and stimulus-independence (of some non-zero degree) with respect to C defines a suppositional space for S with respect to C. This is to say that S can exercise any representational ability R in the class without R's being directly triggered by ‘external’ stimuli.

This gets us some way. But a system in possession of a suppositional space with respect to some class of representational abilities is not yet capable of practical deliberation. To see why, imagine that such a system achieves stimulus-independence by way of a randomly operating internal representation-trigger. And imagine that this system exhibits systematicity by way of randomly associative recombinatory abilities. This system possesses a suppositional space with respect to the representations that the trigger is able to generate, but the activity that takes place in the space does not look anything like practical deliberation. If we had to describe it, we might say that, in the absence of further structure within the suppositional space, such a system is capable only of involuntary mind-wandering. The representations it generates, and the ways it manipulates these representations, will not form a coherent pattern.

By contrast, deliberation is goal-driven. As Gilbert Ryle puts it, ‘Our mental work has a policy, however nebulous, behind it and a discipline, however mild, in it—else it is not deliberating’ [2000: 343]. In deliberation, an agent attempts to find an action that meets some constraint or set of constraints. What are these constraints? Mele [2003: ch. 9] has argued that deliberation arises in response to uncertainty about what to do. We can add to this the plausible thought that this uncertainty arises when cognitive or perceptual systems offer no immediate route to the satisfaction of some need, or to the achievement of some goal. So, as a first pass, we can say that the goals and needs that give rise to uncertainty are those that provide constraints on the deliberative process.

I think that these goals and needs do initially provide a source of constraint. But, for agents possessed of human-like cognitive sophistication, it is important to recognize that constraints come from elsewhere as well. Consider Dave, deliberating about whether to buy skim or, rather, whole milk. Dave's uncertainty arose when the sight of the wall of milk jugs interacted with two competing desires—a desire to enjoy whole milk with his cereal, and a desire to lose weight. Dave's competing desires provide an initial source of constraint, but there could be other features of Dave's situation that provide constraints as well. For example, Dave promised his wife that he would stop buying whole milk. If Dave were to remember this promise, he would realize another reason to buy skim, and he would need to take account of this newly recognized constraint. But even if Dave failed to remember this, there is a sense in which this constraint is real. If Dave buys whole milk and then he returns home, his wife's facial expression is likely to remind him of his promise, and Dave will chastise himself for failing to remember his promise. Dave might plausibly think that his deliberation was defective since in deliberation he failed to recall an important and obvious constraint.

Let us say that, for any deliberative situation D and deliberating agent J, a practical constraint is any feature relevant to D that, if explicitly represented in J's suppositional space, could (e.g. if combined in the right way with prospective decision alternatives) add to J's propensity to view prospective states of affairs relevant to D as to-be-produced (if possible) or as to-be-avoided (if possible). This characterization gives us a sense of the kinds of feature that agents take to constrain deliberation—desires, goals, needs, promises, commitments, cares, prior intentions, and so on. The size and scope of the list of practical constraints will depend on the cognitive and motivational complexity of the agent in question. For adult human beings, the list is potentially enormous. In many contexts, the list far exceeds working memory capacity. Thus, human agents engaged in deliberation are partially engaged in a search to identify relevant practical constraints, and to separate the good constraints from the bad.[Fn fn0009] ^9^This discussion uncovers a deep connection between (at least practically oriented) thought and agency. If deliberation is to be at all coherent, it must be goal-driven, which is to say capable of being controlled to some degree. Somewhat tacitly, Camp recognizes this connection as well [2009: 288]:

Because thoughts are at least partly constituted by their contents, understanding a thought requires grasping the conditions required for its satisfaction. But if a thinker really does grasp those conditions of satisfaction, as opposed to simply being confronted by the conditions themselves, then its grasp of those conditions should be relatively independent of its current circumstances. Otherwise, the world, and not the thinker, is shouldering the bulk of the representational burden. And if this is so, then that ‘thinker’ really is just a passive reactor.

By way of a discussion of stimulus-independence, Camp connects the ability to understand a thought with the ability to exhibit activity with respect to a thought. The view under development presumes that an agent's ability to exhibit activity with respect to a thought requires that the agent possess some control over the deliberative process in which the thought arises.


In addition, agents engaged in deliberation are engaged in the attempt not only to solve some practical problem, but to implement the solution. In acquiring the intention to decide what to do, I take it that agents commit themselves not only to deliberating, but also to terminating deliberation at some point (typically, when good enough reasons are recognized). The following idea—that will become important for my account of deciding—thus emerges from the above discussion. Deciding can be seen as an extension of deliberative activity—as the part that concludes an episode of deliberation.

This gives us an understanding of the nature of deliberation, of how it can be seen as an activity under an agent's control, and some sense of how it is connected to deciding. But we need more than this if we are to understand how a decision would be directly controlled by an agent. In the next section, therefore, I pursue an illuminating analogy between deliberation and skilled overt (that is, bodily) activity.

## 4. Deliberation as Skilled Mental Activity

Randolph Clarke [2010] paints an attractive picture of skilled overt activity, central to which is the notion of motor schemata. According to our best cognitive science of action, motor schemata are internal models or representations of elements of activity that agents develop over time, and that agents learn over time to sequence in various ways. Here is Clarke's description of an instance of skilled overt activity [2010: 531]:

Suppose that Sue is dancing to the Grateful Dead. The dancing is free-form, though the elements composing it are of a relatively small number and are often repeated. The composition of those elements when Sue dances on a particular occasion is improvised, and the particular combination and sequence that make up this instance of dancing is unique. Sue intends to dance now, and she is intentionally dancing. At a certain moment, she swivels her hips just so. That swivel is spontaneous, an unplanned improvisation, but it is nonetheless intentional … As schema theorists would see it, internal models of the components of these various movements reside in Sue's central nervous system. When she dances, her present-directed intention to dance activates in an appropriate sequence the motor commands that, together with her having that intention, produce her movements. This … is a crucial part of what it is for her to be intentionally performing this dance and to intentionally swivel her hips just so.

According to Clarke, Sue's intention to dance plays a crucial role in explaining why her performing a hip swivel is an intentional action. This intention's explanatory work is tied to the causal work it does in initiating, guiding, and sustaining Sue's dancing. But, in addition to the causal work of the relevant intention, Clarke makes room for the role of perceptual feedback. In particular, the present-directed intention is said to ‘evolve in response to … perceptual feedback’ [2010: 530]. Later, Clarke writes: ‘Sue's perception of her environment—of the music and of the locations and movements of other dancers—plays a crucial role in producing the ongoing variation in the movements she makes while she dances’ [2010: 542]. The thought here seems to be that, since exercises of skill often require responsiveness to features of the situation, skilled overt activity often requires that an agent be perceptually attuned to the environment in a certain way. Drawing on Clarke's discussion, then, we can say that an instance of skilled overt activity A crucially involves the following features: (a) relevant motor schemata, the appropriate sequencing of which qualifies as a way of carrying out A, (b) a relevant present-directed intention to carry out A that initiates, sustains, and guides A, (c) a kind of perceptual attunement to the environment that, in collaboration with the ‘evolving’ intention, helps to guide the sequencing of relevant motor schemata. This is an attractive gloss on skilled overt activity, but I have two questions. First, what might it mean for the intention to evolve in response to perceptual feedback? Second, what else might we say about the perceptual attunement at issue?

Regarding the first question, in my view a fruitful way to conceive of an evolving intention is by way of the well-known comparator model of motor control [Wolpert and Kowato 1998; Frith et al. 2000]. According to this model, an intention produces overt action by way of cooperation with a closely coupled collection of modelling mechanisms that take the intention's relatively abstract specification of a goal-state and transform it into various fine-grained, functionally specific, commands and predictions. Thanks to the workings of these closely coupled mechanisms, at multiple stages and at very rapid time scales there are opportunities for the evolution of the intention, in at least two ways. First, feedback might lead to subtle changes to the original intention. For example, Sue notices a change in the music's tempo, and she adjusts her dancing tempo accordingly. Second, feedback might lead to the generation of circumstantially appropriate applications of the intention. As Sue dances, for example, her intention in conjunction with perceptual feedback might lead to the generation of subordinate intentions that accord with the original intention's plan and that can be seen as filling in the details of that plan.

Regarding the second question, it is plausible that the kind of perceptual attunement important in skilled overt activity involves attention. Some argue that attention is necessary for intentional action [Wu 2011]: if that is right, then we get attentional involvement in skilled activity for free. But even if attention is not necessary for intentional action, it is plausible that paradigmatic skilled intentional action displays the fluidity and flexibility it does in part because skilled agents know how to attend to their environments and how to utilize the deliverances of attention as they act.[Fn fn0010] ^10^We know that skilled agents do display different patterns of attention to their environment than novices. For a representative study, see Beilock et al. [2004].


Consider now the suggestion that deliberation is skilled mental activity. On this suggestion, deliberation is viewed as activity initiated, guided and sustained by a relevant intention: the intention to decide what to do. Further, deliberation is constituted by the exercise of mental capacities in deploying various mental operations—mental analogues of motor schemata.[Fn fn0011] ^11^For a view of mental action that uses a similar notion of ‘mental operation’, see Proust [2001]. Call these mental operations deliberative strategies.

Recall that, in the case of overt activity, an agent's behaviour is implemented by way of closely coupled modelling mechanisms that take an intention's relatively abstract specification of a goal-state and transform it into various fine-grained, functionally specific, commands and predictions. This does not mean that all of the implementation of an intention takes place at the ‘sub-personal’ level. A number of ‘personal level’ cognitive operations (especially attentional processes) remain important throughout the course of much overt action, and they influence the way that intentions get implemented, as well as updated and revised [Shepherd forthcoming]. What I am suggesting here is that something similar is true of much mental action. The neuroscientist Masao Ito has argued that, just as the cerebellum houses the internal models of body parts that afford fine-grained motor control, the cerebellum might house internal models of the mental operations performed by cerebral cortex, in so doing affording agents a similar kind of control over their mental operations [Ito 2008]. Ito's case is strengthened by recent work demonstrating that the kind of hierarchical predictive coding exemplified by the comparator model is in fact utilized in a wide range of processes in the brain [Clark 2013]. It is plausible that the kind of closely coupled perceptual, cognitive and actional modelling mechanisms that undergird motor control also undergird control of mental operations.

One might doubt this simply because control over mental action seems more opaque than does control over overt action. Although we clearly have control over a range of mental operations—just try to picture a blue chair and then to rotate it, or try to imagine a song's tune and then to speed up its tempo—we know little about how this control is exercised. But further evidence that control operates similarly in both bodily and mental domains comes from work using neurofeedback. Neurofeedback works like this: you are hooked up to instruments that measure your brain activity (usually via electroencephalography or functional magnetic resonance imaging) and that feed it back to you via auditory or visual feedback. The feedback represents the brain activity and gives you a chance to modulate it, much as you might modulate the movements of your hand, given visual or haptic feedback about its activity. Interestingly, through neurofeedback, human agents can learn to (mentally, voluntarily) control fine-grained features of brain function. Recent studies have demonstrated that, when provided with perceptual feedback of fine-grained brain activity, human agents can rapidly learn to voluntarily control, for example, beta and theta band activity in the cortex (a general electroencephalogram measure of cortical arousal, see Gevensleben et al. [2009]), spontaneous neural activity in retinotopic visual cortex [Scharnowski et al. 2012], and the firing rates of neurons in medial temporal cortex [Cerf et al. 2010]. Importantly, the success of neurofeedback depends on participants receiving a representation of brain activity in a perceptual format. Thus, what participants are doing in these experimental conditions is utilizing perceptual feedback from the environment to control activity in the brain. If the mechanisms responsible for control over mental operations did not overlap significantly with the mechanisms responsible for control over bodily action, it would be difficult to understand participant success in these conditions.

In deliberation, then, it is plausible that the agent has available schemata for a suite of mental operations (deliberative strategies)—imagining, comparing, hypothesizing, and so on. Thanks to their repeated use, these are operations that agents know how to sequence and deploy ‘at will’, in the sense that these operations can be deployed rapidly in response to an indication that doing so is appropriate. This gives us an understanding of how an agent progresses in a controlled manner as she deliberates: an agent is sensitive to the practical constraints that frame her decision problem, and she utilizes a suite of mental operations in an attempt to find reason to terminate deliberation by making a decision.

We are now ready to extend this view of skilled deliberative activity to cover the event of intention formation.

## 5. Control over Deciding

Notice that we can view an intention formation as another mental operation in the agent's store. Now notice that, as is the case with other deliberative mental operations, it is plausible that an agent knows how to deploy or perform this operation ‘at will’, in the sense that this operation can be rapidly deployed in response to perceptual (or cognitive) indications that doing so is appropriate. I say this is plausible because, in part, it seems that facility with such an operation would prove very useful to an agent. After all, while deliberating, agents are often uncertain regarding the time available to them. In some circumstances, the time available quickly comes to a close—an agent realizes that she needs to decide now. It would thus be useful if an agent possessed the ability to terminate deliberation ‘at will’ by forming an intention. Sometimes continued deliberation is costly, and the value of deciding something will trump the value of deciding nothing at all.

But how does an agent terminate deliberation ‘at will’? Return to the thought that skilled overt activity typically involves attentional attunement to relevant particulars of the environment (and the activity), where the particulars in question are dictated by the circumstances, the agent's intentions, the agent's familiarity with components of the activity, and so on. I am suggesting that skilled deliberative activity typically involves attentional attunement to the relevant particulars of the deliberational context. Indeed, regarding intention formation in particular, I want to strengthen the claim about attention: in my view, attention is necessary for the deliverance of the indication that forming a relevant intention is appropriate.

Work on the relationship between attention and demonstrative thought affords a suggestive analogy. A number of theorists maintain that attention is necessary for demonstrative thought (most prominently, Campbell [2002]). Joseph Levine asks how a demonstrative thought—‘that fly’—makes contact with the fly. His answer involves attention [2010: 178]:

Obviously visual perception is what makes the fly available for me to think about. But while perceiving the fly, in this case, is necessary for demonstrating it in thought, it's clearly not sufficient. After all, at the same moment I perceive the fly I also perceive a host of other objects. What's needed in addition to perceiving the fly is attending to it.

The idea is that without attention—here conceived as a process that selects or highlights some item for further processing—there would be no way for the action of mental demonstration to refer directly to the object demonstrated. In my view, a similar argument supports the necessity of attention for deciding. When deliberating, an agent is trying to solve a question about what to do. Various plans for action are available to her in thought, and at the moment of decision she performs the mental operation of intention formation in response to recognition of an indication that doing so is appropriate. How is it that she recognizes the indication? It must be because the plan she adopts (or a reason(s) that favours it) is, at the time, the object of attention.

On the view I am developing, we can see events of intention formation as mental analogues of skilled overt movements such as Sue's hip swivel. The hip swivel is produced when attention-mediated (perceptual) feedback causes an adjustment to Sue's intention to dance: when this causes an adjustment in how Sue's intention guides the sequencing of various motor schemata, or in the generation of subordinate intentions or motor commands that fill in the details of the original intention's plan. Analogously, an intention formation is produced when attention-mediated (perceptual or cognitive) feedback causes an adjustment in how the intention to decide what to do guides the sequencing of various mental operations, or in the generation of the mental operation of intention formation that fills in the details of the original intention's plan.

There are at least three ways in which one might envision this happening. First, the intention to decide what to do could itself be adjusted into an intention to form the relevant intention. One reason to favour this possibility is that, arguably, the intention to decide what to do includes a commitment to terminate deliberation at some point. Perhaps, then, thinking of the relevant intention in this more specific way allows us to see the moment of intention formation as an event built into the intention's plan, much as an event of turning off the High Street onto Catte Street might be an event built into an intention to walk to the King's Arms. Yes, perhaps. But I am dubious. It is much more plausible to think that the progression from an intention to decide what to do to an intention to do something particular involves the satisfaction of the older intention and thus the birth of a new intention.

Second, recognition of the relevant indication, in conjunction with the intention to decide what to do, could generate the acquisition of a subordinate intention with content something like ‘form this intention now’. Notice that this is similar to McCann's view that deciding involves an intention to decide as one does. But this view has the advantage of avoiding any commitment to a mysterious notion of intrinsic or non-relational intentionality. Accordingly, this view is consistent with standard event-causalist approaches to intentional action. Even so, one might worry that this view is uninformative and perhaps *ad hoc*. Is it plausible to posit an intention to form an intention? The kind of process referred to might be better thought of as a sub-personal, non-intentional, mental process.

Third, the intention to decide what to do could directly generate the formation of the new intention. One might worry that this possibility is as uninformative as the last. In this connection, it is worth noticing that the discussion in sections 3 and 4 of this paper makes plain that the intention to decide what to do plays a causal role in an agent's deliberation, in coordination with a wide range of mental operations. The causal work of the intention guides an agent's progress towards a decision, but introspection, imagination, perception, etc. also have a structuring influence on the process. A big part of the puzzle about deciding concerns how a decision comes to have the particular content it does: how could the decision get its content when the intention that generates it does not possess this content? Here is a proposal. The decision inherits its content from the attention-mediated recognition of the indication that deciding to A is appropriate. On this proposal, the intention to decide what to do directly generates the formation of the new intention, and what it generates inherits the content of the plan indicated in the event of recognition.[Fn fn0012] ^12^Thanks to an anonymous referee for prompting me to consider more carefully the difference between these three possibilities.


We have been looking for an account of direct control over decisions that does justice to the view that momentary events of intention formation are intentional actions. According to the account on offer, (non-intentional) intention acquisitions are distinct from (intentional) intention formations, in part because the latter is an active expression of an agent's skilled deliberative activity. Moreover, (intentional) intention formations result from the causal work of a relevant intention, in conjunction with the agent's attentional attunement to relevant features of the deliberative situation. By contrast, (non-intentional) intention acquisitions need not be connected in the same way to intentions to decide what to do, and need not involve attention. Typically, agents acquire intentions when there is no uncertainty as to what to do—deliberative events are not their immediate causal predecessors.[Fn fn0013] ^13^Sometimes, agents acquire intentions when there is no prior uncertainty as to what to do: the intention is immediately acquired. But sometimes the agent deliberates for a while, and then forms a judgment about what it is best to do. It is plausible to think that, in the absence of competing motivation, a default causal connection relates these judgments to events of intention acquisition [Mele 1992: 230]. Thus, sometimes one passively acquires an intention because one's judgment puts an end to one's uncertainty, and causes the intention to be acquired.
^,^ [Fn fn0014] ^14^Am I saying that if an intention acquisition does not involve prior deliberation then the event of acquisition is not an intentional action? Yes: this is a bullet I believe I must bite (thanks to an anonymous referee for pressing me on this point). However, I think that I can put some sugar on that bullet by noting that prior deliberation does not require any particular length of time. The deliberative process can be quite quick. Consider, for example, a Buridan's Ass case in which an agent notices two options and decides very quickly to A rather than B: it is tempting to describe this as a case in which an agent decides ‘immediately’, i.e. with no prior deliberation. But it is arguable that such a decision is preceded by very brief recognition that A and B are very similar, or (roughly) equally desirable, and by this recognition causing momentary uncertainty followed by quick recognition that it does not matter which plan one chooses. On this description, an agent might thereby simply acquire (in a non-actional way) an intention to A. Or she might attend to A, take her recognition that the decision does not matter as indication that deciding to A is appropriate, and in light of this decide intentionally to A. Thus, intention formations need not be seen as ‘just reflex’ or as ‘automatic culminations’ of deliberative activity. The decision is an intentional action when the initiation of the relevant mental operation is the result of the agent's executing the intention to decide what to do, while remaining attentionally attuned to relevant particulars of the activity and thus recognizing an indication to form a given intention as an appropriate indication.

In my view, the above justifies rejection of a non-actional view of deciding. But I cannot resist offering another consideration in favour of this verdict. Notice that proponents of a non-actional view do not typically deny that the deliberative activity that precedes deciding is full of intentional mental action. Strawson, for example, maintains that the deployment of what I have called deliberative strategies—Strawson mentions ‘setting one's mind at the problem’, ‘dragoon[ing] one's wandering mind back to the previous thought content’, initiating ‘a kind of actively receptive blanking of the mind’, and more [2003: 231–2]—is often a matter of intentional action. Why, then, maintain that the formation of an intention cannot be an intentional mental action as well? In my view, the best reason stems from consideration of the control problem I elucidated in section 2. But, with this worry removed, proponents of a non-actional view must confront a tension. If they can countenance deliberative activity as (sometimes) a matter of intentional action, then they need a special reason to deny that events of intention formation deserve the same treatment. I doubt that a convincing one exists.

## 6. Conclusion

Most see practical decisions as intentional actions for which agents can be held responsible. In this paper, I articulated a problem for this commonsensical view. Since an agent engaged in deliberation has no definite idea of what to decide, or of what a good decision will look like, it is unclear how agents can be thought to exercise direct control over their decisions. I have argued that an exercise of direct control over decisions is an extension of the skilled mental activity of deliberation, necessarily involves attention, and is initiated in response to attention-mediated indication that terminating deliberation by forming some intention is appropriate.

None of this makes deciding any easier, of course. When deliberating and deciding, agents face fundamental problems: ignorance of the future and of relevant practical constraints, limited processing capacity, and so on. Even so, it seems that, in deliberating and deciding, agents sometimes manage to exercise control actively over the way things go, and by extension over the course of their own lives. The account of direct control developed here helps us to understand how this is so.[Fn fn0015] ^15^Thanks to two anonymous referees for helpful comments on an earlier draft. Thanks as well to Alison Fernandes, Al Mele, and Till Vierkant for helpful conversation and comments. Finally, thanks to the audience at the 2014 Joint Session of the Aristotelian Society and the Mind Association.

